# A patch-based super resolution algorithm for improving image resolution in clinical mass spectrometry

**DOI:** 10.1038/s41598-019-38914-y

**Published:** 2019-02-27

**Authors:** Klára Ščupáková, Vasilis Terzopoulos, Saurabh Jain, Dirk Smeets, Ron M. A. Heeren

**Affiliations:** 1 Maastricht MultiModal Molecular Imaging institute (M4I), Maastricht, 6229 ER The Netherlands; 2 Icometrix, R&D, Leuven, 3000 Belgium

## Abstract

Mass spectrometry imaging (MSI) and histology are complementary analytical tools. Integration of the two imaging modalities can enhance the spatial resolution of the MSI beyond its experimental limits. Patch-based super resolution (PBSR) is a method where high spatial resolution features from one image modality guide the reconstruction of a low resolution image from a second modality. The principle of PBSR lies in image redundancy and aims at finding similar pixels in the neighborhood of a central pixel that are then used to guide reconstruction of the central pixel. In this work, we employed PBSR to increase the resolution of MSI. We validated the proposed pipeline by using a phantom image (micro-dissected logo within a tissue) and mouse cerebellum samples. We compared the performance of the PBSR with other well-known methods: linear interpolation (LI) and image fusion (IF). Quantitative and qualitative assessment showed advantage over the former and comparability with the latter. Furthermore, we demonstrated the potential applicability of PBSR in a clinical setting by accurately integrating structural (i.e., histological) and molecular (i.e., MSI) information from a case study of a dog liver.

## Introduction

Recent progress in sample preparation protocols, instrumentation, and data analysis strategies have greatly improved the field of mass spectrometry imaging (MSI)^1–3^, making it a powerful tool for untargeted, rapid detection, localization, and simultaneous identification of potentially thousands of molecules from complex sample surfaces^4–6^. Several MSI technologies exist, encompassing a variety of ion sources and mass analyzers suitable for diverse molecular analysis, with each source-analyzer combination enabling different mass and spatial resolutions. Owing to the soft ionization, dynamic mass range, noise tolerance and recent advancement in speed and spatial resolution, Matrix Assisted Laser Desorption Ionization (MALDI) Time of flight (ToF) technology is the most commonly used MSI tool in clinical settings. However, despite the emerging popularity of MALDI-ToF in clinical environments, the lower spatial resolution and limited anatomical information it provides relative to classic histology continue to preclude the technique from standard implementation. The current spatial resolution limit in commercial MALDI-ToF systems is about 10 *μ*m. This resolution is sufficient for detecting whole eukaryotic cells (10–100 *μ*m). Yet, it is inadequate for examining subcellular structures (e.g., nuclei, which vary in size between 0.01 and 10 *μ*m). Moreover, the structural definition pronounced with histology is more obscured at these resolutions in MS images, diminishing the significance of the molecular distribution findings particularly across tissue boundaries.

Contrary to MSI, high resolution optical microscopy involving immunohistochemistry or histological staining offers superior spatial resolution, on the order of 100 nm, and rich anatomical information deriving from finer structural detail. This methodology is widely used by pathologists to examine the fine detail in the composition, organization, and structure of cells and tissues. However, on its own, histology is unable to relate much information regarding the molecular distribution within tissue, which is instead the major strength of MSI. MSI and histology are therefore complementary analytical tools for examining tissue organization and function^4,7^, and both are essential to elucidating our understanding in pathology and systems biology.

Super resolution is a term describing data processing methods that construct high resolution images from observed lowresolution images. It has been successfully applied in many image processing fields, including surveillance, forensics, medical, and satellite imaging^8^. Ever since Tsai and Huang^9^ first introduced the super resolution concept in 1984, many advanced techniques have been developed^8,10^. Super resolution methods can be classified broadly into two different categories: (a) methods that focus on reducing the image acquisition time using parallel imaging and sequence modification^11^ and (b) methods that post-process acquired low resolution images. The latter are generally of interest as they reduce the high instrumental cost, relatively long acquisition time, and the physical limitations of resolution enhancement through hardware. These methods are based either on frequency^8,10,12^ or spatial domain information^11^. Since not all MSI techniques provide frequency information, but all retain spatial information, this work is focused on spatial domain super resolution approaches. Patch-based super resolution (PBSR) is a method where spatial features from a high resolution modality are used as references to guide the reconstruction of low resolution images from the second modality using image redundancy^11^. It finds a similar patch in an image and then attempts to reconstruct pixels by using information from similar neighboring pixels. In a parallel clinical setting, PBSR has previously been applied in the medical imaging field, where low resolution magnetic resonance spectroscopy images were reconstructed in the spatial domain using high resolution magnetic resonance images as reference.^11^. However, to date, there is no known or published record of PBSR applied to MSI data.

In this paper, we used a PBSR algorithm developed in-house to increase the resolution of mass spectrometry images using histology images. We validated the algorithm’s performance using a phantom image, clinically relevant samples of mouse cerebellum, and a dog liver biopsy. We compare its performance against a high resolution MSI reference and two well-known super resolution methods: linear interpolation and image fusion^13^. Additionally, image quality and accuracy were evaluated qualitatively using a checkerboards display and quantitatively by Structural Similarity Index and statistical differences.

## Results

### Comparison of PBSR with other super resolution methods

Performance of the PBSR algorithm was evaluated against two accepted super resolution methods: linear interpolation (LI) and image fusion (IF). The first method, LI, is a rather simple mathematical operation and the most suitable method for the PBSR to be compared with since it is also a weight-based method. The second method, IF, is based on a linear regression analysis scheme developed and validated for upsampling of MSI images with the use of histology.

#### Qualitative evaluation

**Figure 1:**
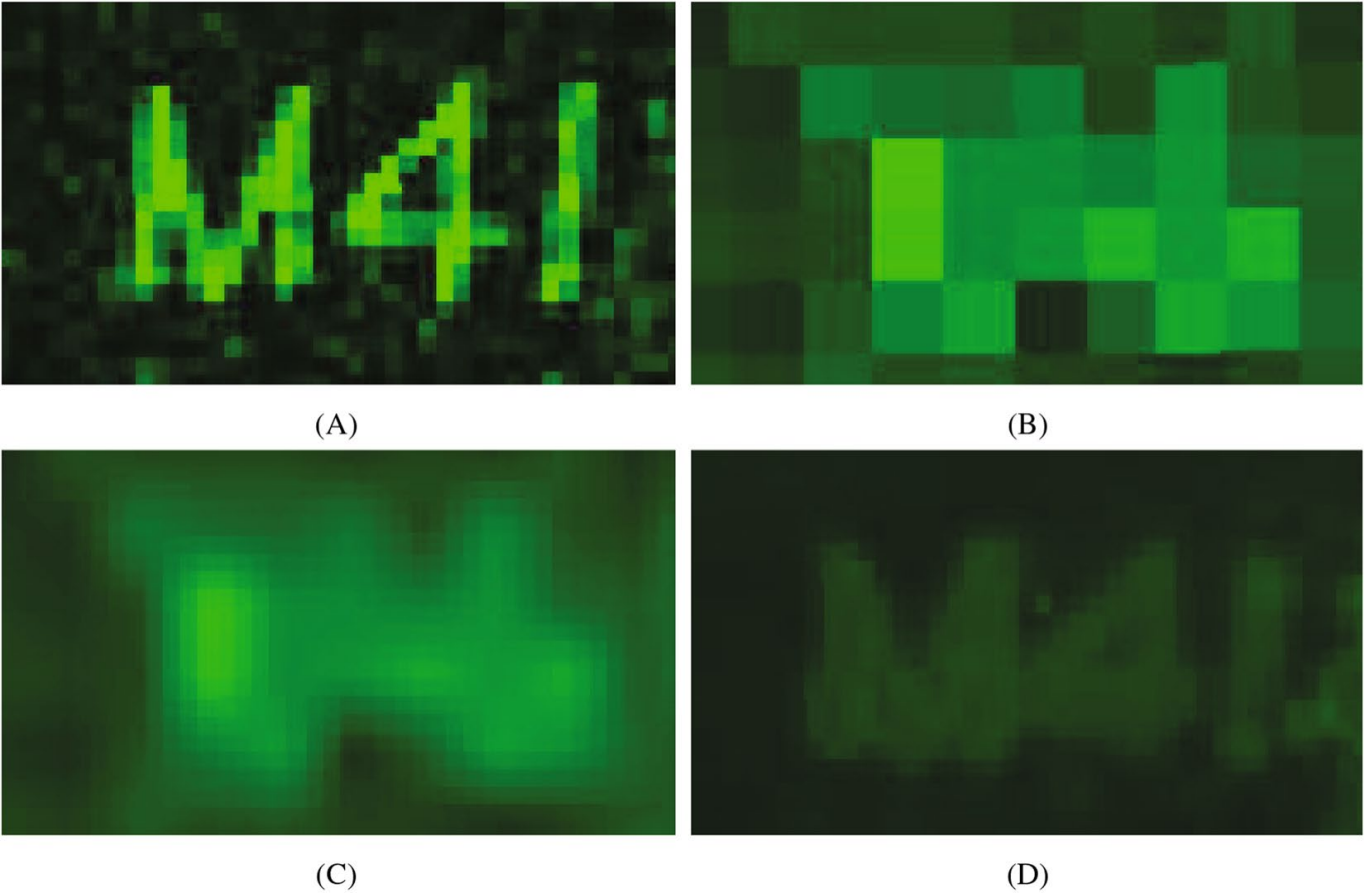
Comparison of the phantom logo images for an m/z 333.2 created by: (**A**) the high spatial resolution MSI (10 *μ*m), (**B**) the low spatial resolution MSI (40 *μ*m), (**C**) the LI upsampling result, and (**D**) the PBSR upsampling result.

**Figure 2:**
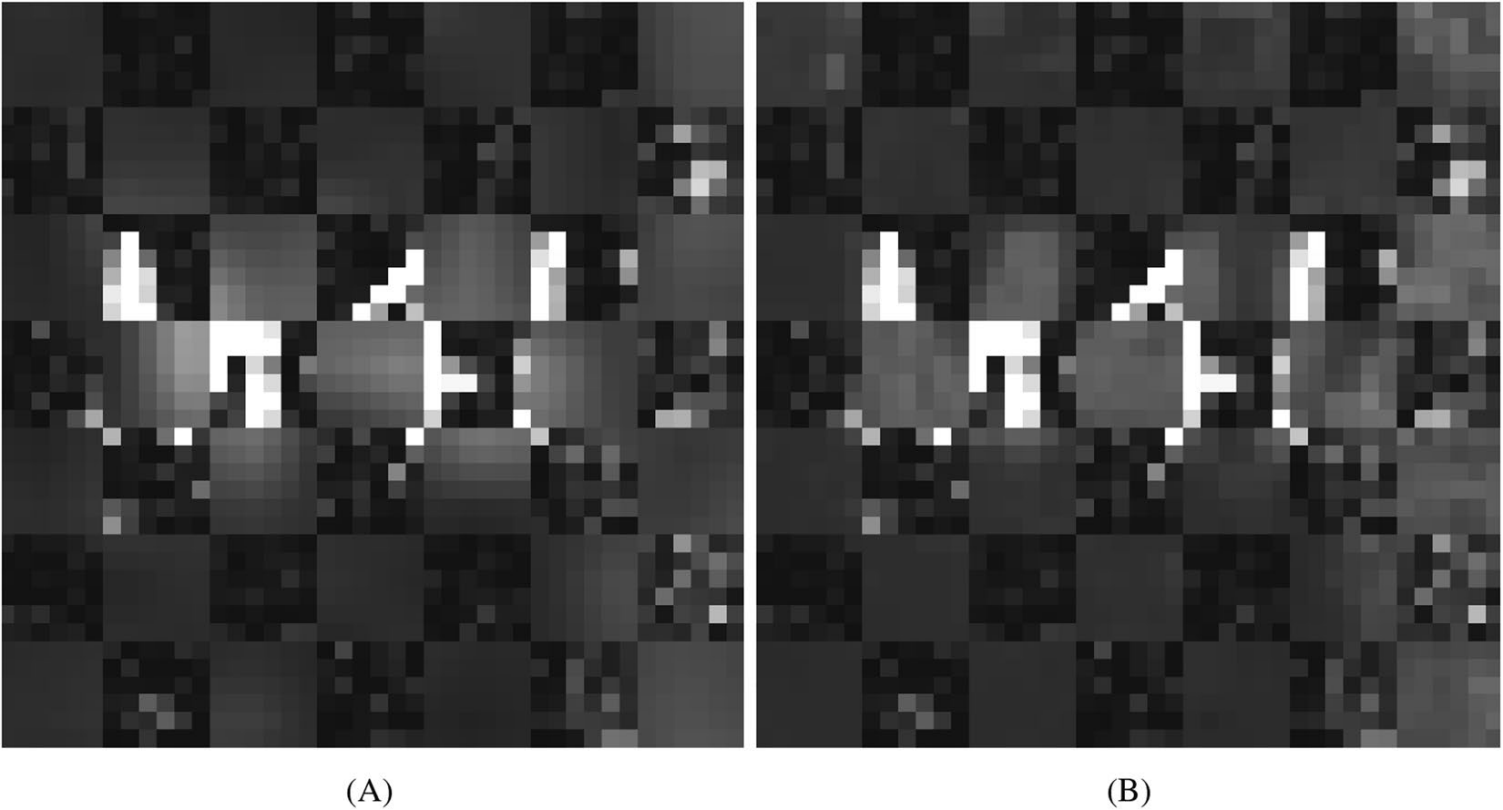
Checkerboard display the reference MS image (10 *μ*m resolution) laid over the (**A**) LI and (**B**) PBSR upsampled images.

Firstly, we applied the PBSR algorithm to a phantom logo. Figure 1a–d show the MS extracted ion images for a matrix peak (m/z 333.2) acquired at a high 10 *μ*m spatial resolution, lower 40 *μ*m spatial resolution, and the upsampled combinations of the low and high resolution image data generated using LI and PBSR. The edges of the logo appear sharp in the high resolution MS image while the low resolution MS image fails to capture them. LI upsampling was not observed to improve the edge sharpness, whereas with the PBSR algorithm the improvement is evident. The images in Fig. 1 are displayed using a green color palette to assist visualization. We also show checkerboard images of the LI and PBSR cases using the high resolution MSI as a reference (Fig. 2a,b). When examined carefully, PBSR provides a better approximation of the real MS reference image.

**Figure 3:**
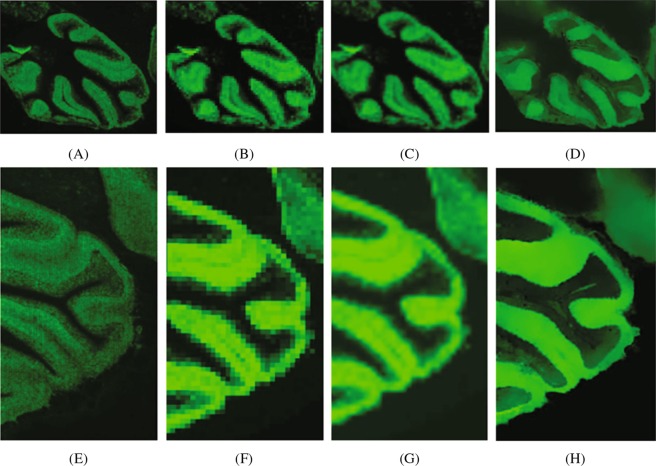
The brain studies comparison. Top row shows brain 1 images for an m/z 790.7 created by: (**A**) high spatial resolution MSI (20 *μ*m), (**B**) low spatial resolution MSI (80 *μ*m), (**C**) LI result, and (**D**) PBSR result. Bottom row visualizes same m/z for brain 2 results: (**E**) high spatial resolution MSI (10 *μ*m), (**F**) low spatial resolution MSI (80 *μ*m), (**G**) LI, and (**H**) PBSR results.

We next assessed the PBSR algorithm’s performance on MS images of mouse brain tissue sections. Brain 1 was acquired at low and high resolutions of 80 *μ*m and 20 *μ*m, respectively. Likewise, brain 2 was measured at low and high resolutions of 80 *μ*m and 10 *μ*m. For both brains, MS images were acquired at the same low resolution but different high resolutions to evaluate the performance of the PBSR algorithm when upsampling to different resolutions. Figure 3a–h shows images for the phosphatidylcholine 36:1 ion acquired with low and high MS spatial resolutions and the corresponding LI and PBSR images obtained when upsampling the 80 *μ*m MS image using either the 10 *μ*m or 20 *μ*m high resolution image as a reference.

**Figure 4:**
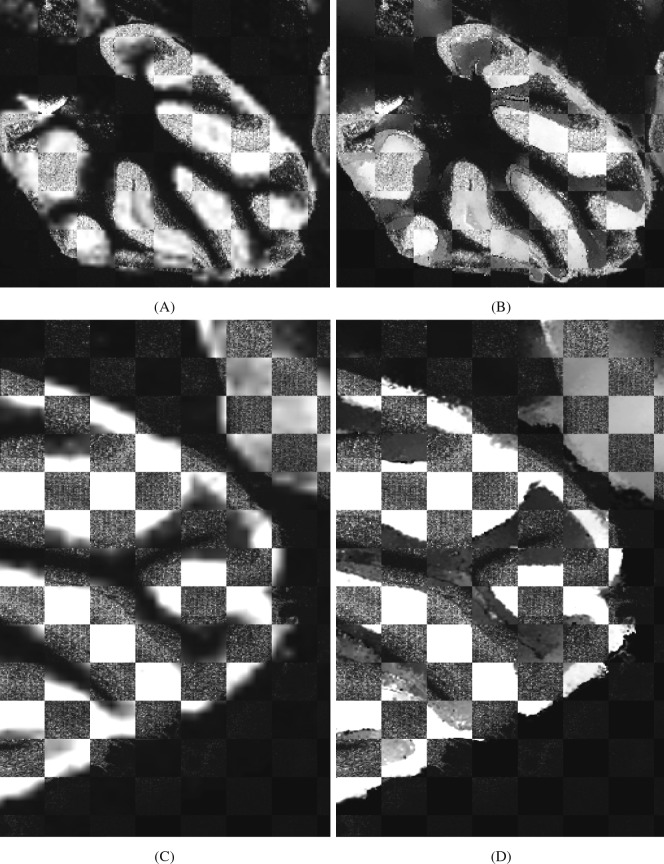
Checkerboard display. Top row: brain 1 reference MS image (20 *μ*m) overlaid with upsampled image by LI (**A**) and PBSR (**B**). Bottom row: brain 2 reference MS image (10 *μ*m) laid over by LI (**C**) and PBSR (**D**) upsampled images.

Checkerboard reference images of brain 1 or brain 2 alternating with the corresponding super resolution methods were also generated and are shown in Fig. 4a–d. It can be observed that the matching between the checkerboard images overlaying the reference MS images and the PBSR results are satisfactory in both brain data sets.

**Figure 5:**
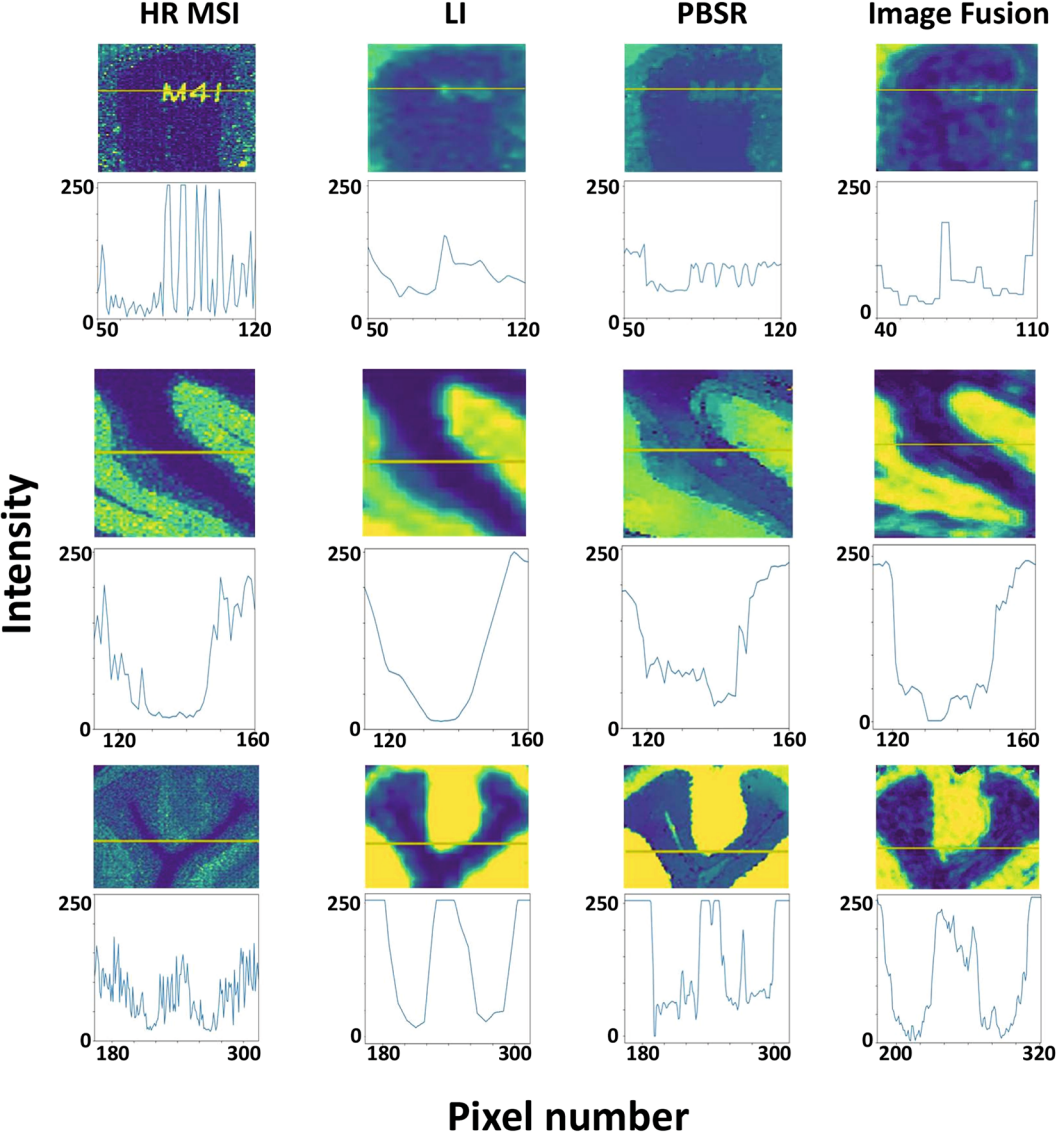
Intensity profiles. Top row: phantom logo, middle row: brain 1, bottom row: brain 2. First column shows high spatial resolution MSI, second column shows LI upsampling result, third column shows PBSR results and last column displays IF. The yellow lines represent the pixel rows across which the intensity for each pixel was plotted.

Final qualitative test of the PBSR algorithm’s performance was done by plotting the intensity profiles across the reference MS image and the super resolution results. This time, the PBSR algorithm is also compared with the IF tool presented by Van de Plas *et al*.^13^. Figure 5 shows the intensity profile across the phantom image (logo), brain 1, and brain 2 for the reference high resolution MS image and for the LI, PBSR, and IF results. In the case of the phantom image, the sharp letter edges of the M4I logo clearly defined in the high resolution MS reference image are represented by sharp peaks in the intensity line profile (Fig. 5, top row). This sharpness and detail was lost in the LI’s intensity profile which captured only the overall change in intensity trend. The intensity profile of the PBSR and IF showed improved sharpness and edge detection displaying more sensitive intensity profiles. Similar observations were obtained with both brain studies. LI images could only reproduce broader trends in the intensity profile, whereas PBSR and IF images were more successful at retaining edge definitions and capturing smaller variations in intensity across tissue boundaries.

#### Quantitative evaluation

**Figure 6:**
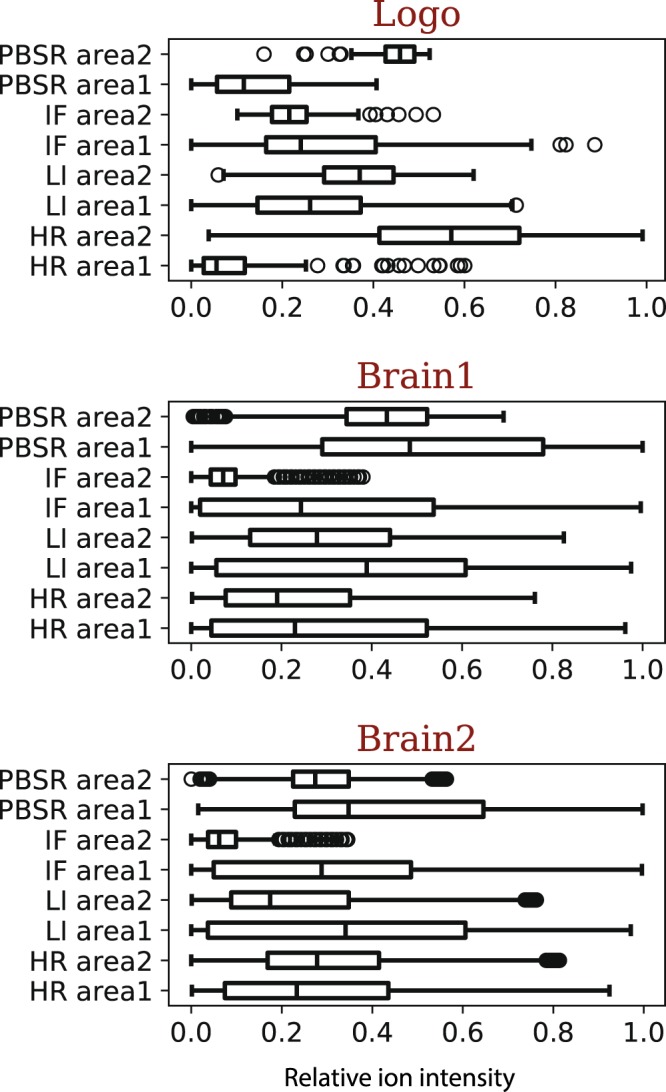
Boxplots displaying the relative ion intensity of pixels in area 1 and area 2 in on Logo (top), Brain 1 (middle) and Brain 2 (bottom) datasets of all methods. Area 1 = intensities in the surrounding tissue pixels around area 2, Area 2 = pixels intensities within the ROI. Area 1 and area 2 are reported as median (first quartile - third quartile). The whisker displays mean +/− 2.7 standard deviation and the dots represent outliers. PBSR = patch-based super resolution, IF = image fusion method, LI = linear interpolation, HR = acquired high resolution image.

Quantitative performance evaluation was done by Structural Similarity Index (SSIM) and statistical measures of difference. The SSIM was calculated for LI and PBSR results only, and showed rather inconsistent behavior (Table 1). While the SSIM computed for the phantom study (logo) showed higher values for PBSR than LI (0.37 vs 0.34), the reverse case was true comparing SSIM values calculated for brain 1 (0.81 vs 0.88) and brain 2 (0.41 vs 0.42). Given that the global image assessment yielded incoherent results, we performed an additional statistical difference measure on a small subset of each image. Area 1 was defined as the pixel intensities in tissue regions surrounding area 2, where area 2 represents the pixels intensities within the tissue region of interest (ROI). This statistical evaluation of the PBSR algorithm compared with LI and IF results was performed using Welch’s *t*-test^14^ together with effect size calculation by Cohen’s *d*^15^. Figure 6 displays the boxplots of ion intensities within area 1 and area 2 for phantom study (logo), brain 1 and brain 2. In the phantom study, we observe that all methods have significant *p*-values (<0.001) with the PBSR method retaining the best contrast (*d* = 3.21), followed by LI (*d* = 0.56) and IF (*d* = 0.4) methods. This advantage was evident in the phantom logo image both visually as well as quantitatively (see Table 1 & Fig. 6 top panel). Examining super resolution image results for brain 1 and brain 2, it was more difficult to visually distinguish the difference (Fig. 6, middle & bottom panel), however, quantitatively, the contrast could be computed (Table 1). For brain 1 all methods have significant *p*-values (<0.001), with the IF method’s effect size being large (*d* = 1.04), followed by a medium effect size for PBSR (*d* = 0.40), LI (*d* = 0.36) and HR (*d* = 0.26). Similarly for brain 2, *p*-values were significant (<0.001) for all methods, with the IF method’s effect size (*d* = 1.22), PBSR (*d* = 0.70), LI (*d* = 0.44) and HR (*d* = 0.11).

**Table 1:** Quantitative measures for measuring the accuracy of all methods on Logo, Brain 1 and Brain 2 datasets.

	Area 1 (surroundings)	Area 2 (tissue)	Effect size	SSIM
**Logo**				
HR	0.06 (0.03, 0.12)	0.57 (0.41, 0.72)	−2.36	*NA*
LI	0.26 (0.15, 0.37)	0.37 (0.29, 0.44)	−0.56	0.34
IF	0.24 (0.16, 0.41)	0.22 (0.18, 0.25)	0.4	*NA*
PBSR	0.12 (0.06, 0.22)	0.46 (0.43, 0.49)	−3.21	0.37
**Brain 1**				
HR	0.23 (0.04, 0.52)	0.19 (0.08, 0.35)	0.26	*NA*
LI	0.39 (0.06, 0.61)	0.28 (0.13, 0.44)	0.36	0.88
IF	0.24 (0.02, 0.54)	0.07 (0.04, 0.10)	1.04	*NA*
PBSR	0.48 (0.29, 0.78)	0.43 (0.34, 0.52)	0.4	0.81
**Brain 2**				
HR	0.23 (0.07, 0.44)	0.28 (0.17, 0.41)	−0.11	*NA*
LI	0.34 (0.04, 0.61)	0.17 (0.09, 0.35)	0.44	0.42
IF	0.29 (0.05, 0.49)	0.06 (0.04, 0.10)	1.22	*NA*
PBSR	0.35 (0.23, 0.65)	0.27 (0.23, 0.35)	0.70	0.41

Welsh’s *t*-test *p*-value for testing the statistical difference between area 1 and area 2 was significant (<0.001) throughout all datasets. Area 1 = intensities in the surrounding tissue pixels around area 2, Area 2 = pixels intensities within the ROI, effect size = the magnitude of statistical difference. Area 1 and area 2 are reported as median (first quartile - third quartile). HR = acquired high resolution image, LI = linear interpolation, IF = image fusion method, PBSR = patch-based super resolution, SSIM = structual similarity index, *NA* = not applicable.

### Clinical application of the PBSR: dog liver study

**Figure 7:**
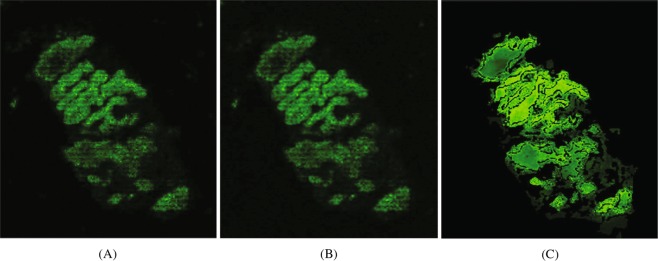
Comparison of the dog liver images for a m/z 906.7 created by: (**A**) original high resolution MSI (10 *μ*m), upsampling results at 2.5 *μ*m resolution of (**B**) LI and (**C**) PBSR.

Encouraged by the proof of concept data sets, we applied the PBSR algorithm to an ongoing study on lipid distribution in liver^16^. The objective was to identify specific lipid markers that define the structural elements of the liver. The fresh frozen dog liver was cryosectioned and prepared for MALDI MSI analysis. MALDI MS images were acquired with a 10 *μ*m spatial resolution, the highest spatial resolution available which still provides sufficient sensitivity. Post MSI analysis, the liver tissue was H&E stained and examined by an expert pathologist. After initial data analysis, an interesting molecular distribution of m/z 906.7, later identified as sulfatide (ST-OH 42:1), was observed near the bile duct cavity. Given that the average cell size ranges from 5 to 100 *μ*m and that we were restricted to a 10 *μ*m resolution, it was not possible to conclude if this sulfatide was only localized within the bile duct and could be therefore used as bile duct epithelium specific marker. Therefore, we applied the PBSR algorithm to the corresponding MS and histological images. The high resolution MS reference image, LI and PBSR results are shown in Fig. 7. From the PBSR upsampled image to the 2.5 *μ*m spatial resolution, it is visible that the ST-OH 42:1 (m/z 906.6618) localizes to the bile duct epithelial layer.

## Discussion

In this study, we evaluated an alternative super resolution method for MSI. Its purpose is to enhance the spatial resolution of MS images to provide confident localization of molecular signals.

The evaluation of the PBSR performance was done both qualitatively and quantitatively. The first measure was visual image quality comparison between the accepted LI and the PBSR algorithm results. The second measure was display of reference high resolution MS image in a checkerboard with each method. The visual assessment and the checkerboard evaluation provided overall image similarity judgment of each upsampling results compared to the MSI reference. In the phantom image, differences between the results of PBSR and LI are visible (Figs 1 and 2). The favorable performance of the PBSR algorithm is explained by the use of the histology image as a guide in the reconstruction process. This helps to discriminate the different tissue types and, hence, results in better sharpness and edge detection. This case is particularly noticeable in the checkerboard display of brain 1 and brain 2 studies (Fig. 4b,d). In the brain studies, the three segmentation masks used were background, white matter, and gray matter. The high resolution MSI of brain 2 (Fig. 3e) shows clear boundaries of the white and gray matter as the cortex folds within the cerebellum. These details are lost in the low resolution MSI as well as in the LI results (Fig. 3f,g). It is the histology guided PBSR algorithm that is able to discriminate the two tissue types (Fig. 3h). Furthermore, the ability to input prior knowledge not only results in better PBSR performance, but it also allows for increased confidence in the upsampling process. When working with clinical samples, the use of histological segmentations is especially beneficial. Expert pathologists consider histopathological annotations as the gold standard and thus are more inclined to accept the PBSR results over other super resolution methods that do not use pathologist provided segmentations. Of course, the prior knowledge might not always be available. In that case, the PBSR pipeline is able to run without segmentations, however, the performance is not ideal (data not shown).

The third qualitative measures applied were intensity profiles plotted across the different super resolution results (PBSR, LI, and IF) and the true high resolution MS image. The intensity profiles evaluate each method’s resolving power in terms of its sensitivity to changes in intensity pixel by pixel. Unfortunately, IF results required manual alignment with the high resolution MSI, which was very time consuming, and therefore unfeasible. The intensity profiles required only a small area of the image to be aligned. Therefore, we restricted ourselves to include the IF results for the comparisons that use only sub area of the images. In these profiles an intensity value per pixel is plotted across the image showing how well changes in intensity are captured. This measure provides information on the sharpness of the features within the images. The intensity profile of the phantom reference image (Fig. 5, top row) clearly shows several intensity drops and rises as we cross the letters of the logo. While the overall trend of increase and decrease in intensity is captured by the LI and IF, the intensity profile of the PBSR result is the closest to the profile of the reference image. Similar conclusions can be drawn from the intensity profiles of brain 1 and brain 2 studies (Fig. 5, middle and bottom row). LI only retains the global trend, whereas the PBSR algorithm and IF are more sensitive in picking up the smaller intensity changes. The explanation is, again, the use of the high resolution histology images that guide both upsampling processes in the PBSR & IF. In contrast, the lack of the complementary information from the histology images and the simplistic nature of the LI method results in loss of information and thus exhibits inferior performance. When comparing the intensity profiles of PBSR with the reference high resolution MS image, differences can be observed. This mismatch can be explained by: (a) noise in the MS reference image vs the smoothed PBSR images guided by the use of the clear segmentations, (b) inherent limitation of the PBSR method which cannot be expected to recover small-scale features that were not sufficiently picked up by the low resolution MSI.

The biggest challenge we encountered in this study was finding an optimal method for quantitative validation. Evaluation of image similarity across different resolutions is not straightforward. Firstly, the current state of the art MSI does not allow absolute quantification. Second, if one chooses to obtain the low and high resolution MSI data from same tissue, one cannot use the same instrument settings in both resolutions. The resulting data differ in the dynamic ranges of all the intensities detected from the different MSI resolution experiments. Thus, they cannot be directly compared. The reason is that the relation between the 80 *μ*m and 10 *μ*m ablation is non-trivial, non-linear and laser specific. Even if one chooses to obtain the MSI data at the same resolution from two subsequent tissue sections, direct comparison using a numerical pixel by pixel measure is not feasible. That is because there are physiological differences between the subsequent tissue sections. These physiological differences would be classified as prediction errors, making it impossible to differentiate genuine prediction error from neighboring section-related differences. We were interested to preserve the cellular anatomical features and their biochemical composition. Hence, we chose to obtain both MSI data sets from the same tissue section.

Despite all these hurdles, we performed SSIM and statistical measures to quantitatively evaluate the PBSR performance. Both of these methods were previously used to evaluate the PBSR performance for details see Jain *et al*.^11^. The SSIM assesses the global image similarly pixel by pixel. Therefore for aforementioned alignment issues, this measure could not be performed on the IF results as even a small misalignment would have considerable effect on the computed SSIM score. In the case of the phantom study the SSIM score for the PBSR algorithm is higher (Table 1), showing better performance than LI. Opposite to that, the brain 1 and brain 2 SSIM scores favor the LI over the PBSR method. This incoherence of the SSIM score for the PBSR algorithm can be attributed to many reasons. One plausible explanation follows. The reference MS image is rather noisy, whereas the use of segmentation masks in the PBSR algorithm results in smoothed images. It appears that this noisiness is preserved by the LI method and thus when SSIM compares pixel to pixel similarity, the image that retains the noise is judged as superior. While the PBSR algorithm results in images with better contrast and smooth edges, it could be the lack of the noise in the images that results in bigger pixel to pixel error and therefore overall low SSIM score.

Since this noise issue is really prominent in the numerical pixel to pixel evaluation of the global image similarity, we opted for the statistical difference measure to be only confined to a sub area of the images. Also, the restriction to only sub areas of images allowed us to include the IF results that were manually aligned. The statistical difference was computed using Welch’s *t*-test^14^ and the effect size was calculated with Cohen’s *d*^15^. Figure 6 displays boxplots for ion intensities of area 1 and area 2. The *p*-value, describing the significance of ion intensity difference between area 1 vs area 2, is reported together with the magnitude of this difference *d* in Table 1. Throughout all datasets it can be observed that the *p*-values for all methods are significant (<0.001). It is the magnitude of this difference that varies from method to method. For the phantom study the PBSR retains the highest contrast, whereas in brain 1 and brain 2 the IF has the largest effect size. Often the IF and PBSR outperform HR (the reference high resolution MS image) in its effect size. This phenomenon indicates that: (a) the effect of noise in these quantitative evaluations, including the statistical difference measure, is still significant, and (b) the use of histology image in both IF and PBSR results in enhanced contrast which is not present in the HR image.

Both, PBSR and IF, offer better image sharpness than LI and help improve visual feature discrimination. When comparing the PBSR algorithm and IF they both perform well, however, from computational point of view, the PBSR method requires fewer parameters to be optimized. This decrease in analytical complexity results in simplification of the underlying optimization problem. A limitation in our current PBSR pipeline is the semi-automatic registration of histology and MSI. This is suboptimal and therefore future work will focus on fully automatic histology and MSI registration. Another already discussed drawback can be the lack of the segmentations that enhance the PBSR algorithm’s performance.

In this work, we successfully applied the PBSR method to investigate the spatial distribution of liver sulfatide Fig. 7. This sulfatide (ST-OH (42:1), m/z 906.6618) was previously identified^16^ as a potential marker of the bile duct epithelium in both healthy dog and human liver tissue. Flinders *et al*.^16^ used MALDI MSI in combination with high spatial resolution secondary ion mass spectrometry imaging and immunohistochemistry to verify the localization of this sulfatide. Further research showed that this marker has completely disappeared in advance stages of primary sclerosing cholangitis. They indicated that this sulfatide was able to discriminate between healthy liver tissue and tissue from patients with severe primary sclerosing cholangitis. Our study showed that the same conclusions regarding the localization of the sulfatide to the bile duct epithelium can be reached if the PBSR algorithm is applied to the MALDI MSI and histology. That means that the secondary ion mass spectrometry measurement can be omitted, saving time and resources.

In the future, this method can be easily applied to any other MSI technology, increasing its flexibility of use. For example, the aforementioned high resolution secondary ion mass spectrometry could serve as the high resolution modality and could be used to upsample MS images with lower resolution acquired by other MSI technologies. Moreover, these images could be further spatially enhanced by another even higher resolution modality, such as electron microscopy images. This would enable direct comparison, assessment, and correlation of molecular and structural information at the subcellular level, opening unprecedented research opportunities.

In conclusion, we present the PBSR algorithm as an alternative super resolution method for MS images. The qualitative and quantitative assessment showed that super resolution methods that use another modality to guide the reconstruction, PBSR and IF, are more effective. In the PBSR pipeline, prior knowledge in the form of the histological segmentations provides anatomical control in upsampling of the low resolution MS image. The application of the PBSR method to the dog liver study allowed for integration of the structural and molecular information from histology and MSI enabling identification and localization of ST-OH 42:1 (m/z 906.6618) in the bile duct cell layer.

## Methods

### Method description

**Figure 8:**
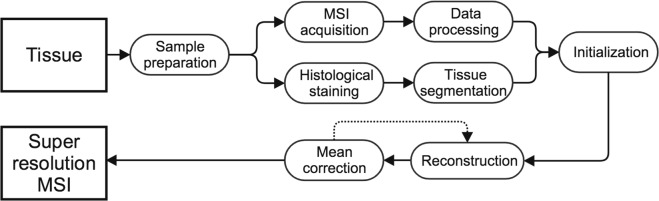
Schematic diagram for upsampling MSI.

Figure 8 presents an overview of our pipeline. The input tissue was processed and thereafter upsampled using the PBSR method. The processing steps consist of sample preparation, MSI acquisition and data processing, histological staining and tissue segmentation. The PBSR pipeline involves initialization, reconstruction and mean correction steps. We describe these steps in detail as follows.

#### Sample preparation

All fresh frozen tissues were cryosectioned with a thickness of 10 *μ*m, at −18 °C on a Cryostat HM525 (Microm, Walldorf, Germany) and thaw mounted onto indium tin oxide coated glass slides^4^. Afterwards, norharmane matrix (Merck KGaA, Darmstadt, Germany) was sublimed onto the vacuum-dried tissue with a custom-built sublimation device (IDEE, Maastricht University, The Netherlands) to enable the analysis of lipid species by MALDI MSI. Data acquisition was performed with the Bruker RapifleX MALDI TissuetyperTM (Bruker Daltonik GmbH, Bremen, Germany) operating in negative ion reflectron mode with a nominal acceleration potential of 20 kV. Data were acquired at a mass range of 300–1000 Da.

#### MSI data acquisition and processing

All objects were acquired at low and high spatial resolution by MSI. Since preservation of the anatomic features within the tissue was of a great interest, we performed the high resolution MSI on the same tissue section rather than on a consecutive one. The raster pixel size as well as laser scan size were set to the same dimensions, allowing the whole pixel area to be irradiated equally. Sufficient material was left in the tissue after the low resolution MSI experiment to perform the high resolution MSI acquisition. The exact raster pixel size and laser scan size settings for each MSI experiment are presented in the data description section.

The raw data were imported to SCiLS software v.2016a (SCiLS, Bremen, Germany), from there it was extracted and imported to MATLAB (v.R2015). HistDistGUI, in-house developed tool using the ChemomeTricks toolbox^17^ for MATLAB (v.R2015) was used for visualization of the MSI data per m/z of interest. Normalization by the total ion count was performed in all cases.

#### Histological staining and tissue segmentation

Standard hematoxylin and eosin (H&E) protocol was applied according to the supplier’s instructions (Merck KGaA, Darmstadt, Germany). After the staining and drying, the slides were imaged with a microscope using bright field light microscope Leica DM6000B (Buffalo Grove, IL, USA) and MIRAX scanner (Zeiss, Breda, The Netherlands) at a resolution of 0.5 *μ*m. The histological image was manually segmented into classes of interest, the details are presented in the data description section.

#### Initialisation

The PBSR pipeline was initialized by (a) upsampling the low resolution MS image using linear interpolation (with a scale factor of 2), (b) by aligning the histology and MS image. The registration between the MSI low resolution image and the histological image was performed using Elastix^18^. A similarity transformation was estimated (i.e. Rotation, translation and scaling) using three levels of resolution, 1024 iterations and mutual information was used as the similarity metric. For the phantom study and the brain samples, the high resolution MS images were manually registered with the low resolution, using Slicer^19^, (only translation was sufficient), and then were transformed to the histology image space using the estimated transformation from Elastix.

#### Patch based super resolution

Reconstruction step: the ion intensity at each higher resolution histology pixel was estimated using relevant neighboring pixels in the MS image, which were computed using the tissue segmentations along with image intensities of histology image. Mean correction step: this step rectifies the estimated ion intensity in every high resolution pixel by taking into account the error toward the corresponding lower level pixel’s ion value. The method iterated between reconstruction and mean correction steps such that the corrected ion intensity map from previous iteration is used to initialize the ion intensity prior map for the current iteration. The convergence of our method is detected when the relative ion intensity difference between the current and previous iteration is negligible.

### Other methods

We compared our method against linear interpolation and image fusion^13^ techniques. LI is a simple weight-based method that interpolates the new point (high resolution gridpoint) between the old points (low resolution gridpoint). IF uses a multivariate partial least-squares regression model that estimates the parameters related to high resolution MS image using corresponding high resolution optical microscopic maps.

### Data description

The following experiments & methods were approved by the Medical Ethics Board of Maastricht University Medical Center (MUMC+), in line with the ethical guidelines of the 1975 Declaration of Helsinki.

#### Phantom study – M4I logo

An artificial pattern in the form of M4I logo was laser micro dissected into rat brain tissue section providing features for the upsampling. The laser thickness was 14 *μ*m and the dimensions of the logo were 241 × 131 *μ*m. The MSI data was acquired at 40 *μ*m (low resolution) and 10 *μ*m (high resolution). The corresponding histological image was segmented into foreground (tissue), background (outside tissue) and the logo area itself. The histology image as well as the segmentations are displayed in Supplementary Fig. S1.

#### Rat brain samples

Two Adult Wistar Han rat’s brain samples were provided by the animal experiments facility of the MUMC+ in Maastricht, The Netherlands. The MSI data was acquired at 80 *μ*m (low resolution) and 20 *μ*m (high resolution) for the first sample - brain 1 and 80 *μ*m (low resolution) and 10 *μ*m (high resolution) for the second sample - brain 2. The corresponding histological images were manually segmented into foreground (brain), background (outside brain), white matter and gray matter. All histology images and segmentations can be found in Supplementary Figs S2 and S3.

#### Dog liver sample

Finally, a tissue section from dog liver obtained from Janssen pharmaceutical was used to demonstrate the ability of the PBSR method to upsample MS images beyond the MADLI MSI spatial resolution limits. The MSI data was acquired at 10 *μ*m (low resolution). The corresponding histological image was also manually segmented by an experienced pathologist into background, bile duct epithelium and bile duct lumen. Segmentations masks and histology image can be found in Supplementary Fig. S4.

### Performance tests

#### Qualitative

We compare the three methods against reference high resolution MS image by means of qualitative measures: (i) visual image quality assessment, (ii) checkerboard images displaying the reference MS image and upsampled result, and (iii) spatial resolving power. The visual quality assessment compares the visibility and sharpness of features within the different MS and histology images and the upsampled results. Spatial resolving power measures the sharpness of features such as tissue/cell borders within the samples. We assess the spatial resolving power of each method by presenting the intensity profile across a feature of interest.

#### Quantitative

The global image similarity was assessed by Structural Similarity Index (SSIM)^20^. The SSIM score is ranges between 0 and 1, where value of 1 is only reachable in the case of two identical sets of data. This measure is mathematically the most comparable with human visual assessment. Moreover, statistical difference in the ion intensities between area 1 and area 2 in a subset of image are tested using the Welsh’s *t*-test^14^. The magnitude of this difference, also known as the effect size, is calculated using the Cohen’s *d*^15^. For each dataset, area 1 was defined as a neighborhood of 2–3 pixels around area 2. Area 2 consisted of: for the phantom study the M4I logo segmentation mask, for brain 1 and brain 2 the gray matter mask.

## Supplementary information

A patch-based super resolution algorithm for improving image resolution in clinical mass spectrometry

## Data Availability

The datasets generated during and/or analyzed during the current study are available from the corresponding author on reasonable request.
