# Supplementary material for: A patch-based super resolution algorithm for improving image resolution in clinical mass spectrometry
Source: Sci Rep. 2019 Feb 27;9:2915. doi: 10.1038/s41598-019-38914-y (PMC6393664; doi:10.1038/s41598-019-38914-y)
Supplement: Supplementary file 1 — A patch-based super resolution algorithm for improving image resolution in clinical mass spectrometry [file 41598_2019_38914_MOESM1_ESM.pdf]

A patch-based super resolution algorithm for improving image resolution in clinical mass spectrometry

Klára Ščupáková[1,2,*], Vasilis Terzopoulos[2], Saurabh Jain[2], Dirk Smeets[2], and Ron M.A. Heeren[1]

[1]Maastricht MultiModal Molecular Imaging institute (M4I), Maastricht, 6229 ER, The Netherlands
[2]icometrix, R&D, Leuven, 3000, Belgium
[*]Corresponding author: k.scupakova@maastrichtuniversity.nl; r.heeren@maastrichtuniversity.nl

SUPPLEMENTARY INFORMATION

The section below displays the Hematoxylin & Eosin stained histology images acquired at 0.5 μm pixel resolution using the MIRAX scanner post MSI analysis. Furthermore, corresponding segmentation masks that were used by the PBSR method are displayed for phantom logo, brain 1, brain 2 and dog liver, respectively (Figs. 1, 2; 3 and 4).

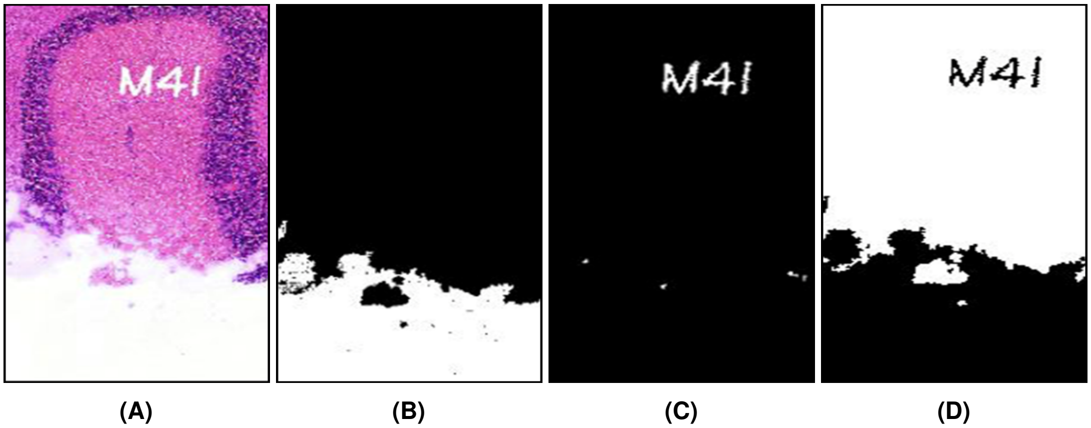

Figure 1. Phantom logo: (A) histology image at 0.5 μm, segmentation masks: (B) background, (C) M4I logo, and (D) foreground.

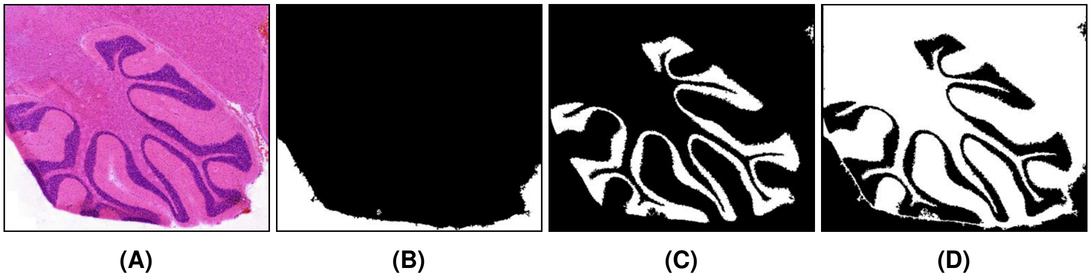

Figure 2. Brain 1: (A) histology image at 0.5 μm, segmentation masks: (B) background, (C) gray matter, and (D) white matter.

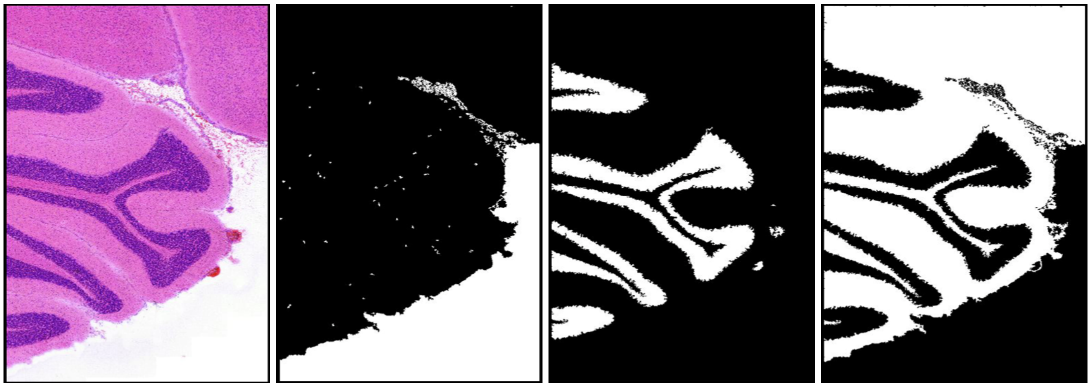

(A) (B) (C) (D)

Figure 3. Brain 2: (A) histology image at 0.5 μm, segmentation masks: (B) background, (C) gray matter, and (D) white matter.

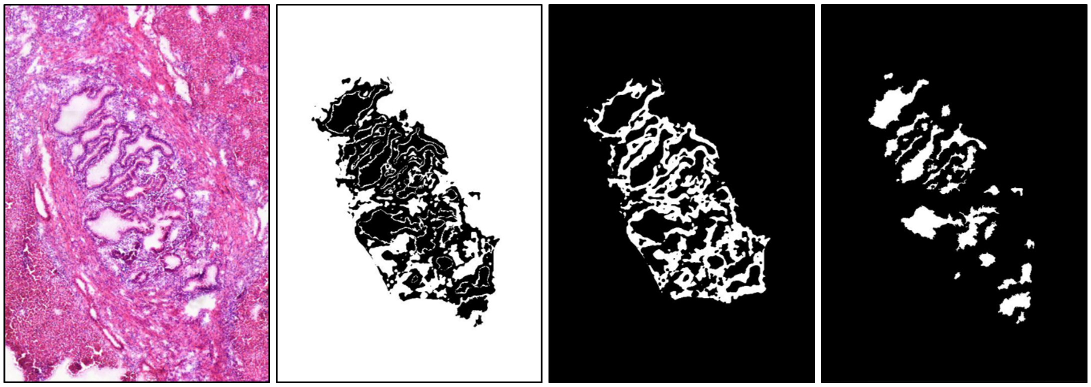

(A) (B) (C) (D)

Figure 4. Dog liver: A) histology image at 0.5 μm, segmentation masks: (B) background, (C) bile duct epithelium, and (D) bile duct lumen.
